# Glycemic fluctuation exacerbates inflammation and bone loss and alters microbiota profile around implants in diabetic mice with experimental peri-implantitis

**DOI:** 10.1186/s40729-021-00360-9

**Published:** 2021-08-17

**Authors:** Hao Li, Yufeng Wang, Dong Zhang, Tsute Chen, Arthur Hu, Xiaozhe Han

**Affiliations:** 1 Department of Prosthodontics, the Affiliated Hospital of Stomatology, Guangxi Medical University, 10 Shuangyong Road, Nanning, 530021 People’s Republic of China; 2 Department of Immunology and Infectious Diseases, The Forsyth Institute, 245 First Street, Cambridge, 02142 USA; 3 Department of Oral Mucosal Diseases, Ninth People’s Hospital, College of Stomatology, Shanghai Jiaotong University School of Medicine, Shanghai, People’s Republic of China; 4 Department of Oral Surgery, Qilu Hospital of Shandong University, Jinan, 250012 People’s Republic of China; 5 Department of Microbiology, The Forsyth Institute, 245 First Street, Cambridge, 02142 USA; 6 Department of Oral Medicine, Infection and Immunity, Harvard University School of Dental Medicine, 188 Longwood Avenue, Boston, 02115 USA

**Keywords:** Peri-implantitis, Glycemic fluctuation, Bone loss, Inflammatory cytokine, TLR signaling, Microbiota

## Abstract

**Background:**

The impact of glycemic fluctuation under diabetic condition on peri-implantitis in diabetic patients remains unclear. We hypothesized that glycemic fluctuation has greater adverse effect on experimental peri-implantitis, compared with sustained high blood glucose in diabetes.

**Results:**

Maxillary left first and second molars of diabetic db/db mice were extracted and were replaced with one dental implant in the healed edentulous space. Glycemic control or fluctuation were managed by constant or interrupted oral administration of rosiglitazone to these mice. Meanwhile, experimental peri-implantitis was induced by ligation around implants. After 14 weeks, inflammatory responses, and peri-implant bone loss, together with oral microbiota profile were analyzed. Diabetic mice with glycemic fluctuation showed greater peri-implant bone loss, inflammatory cell infiltration, and osteoclastogenesis, compared with mice with sustained hyperglycemia. Compared to sustained hyperglycemia, glycemic fluctuation led to further increase in IL-1β, TNFα, RANKL, TLR2/4, IRAK1, and TRAF6 mRNA expression in peri-implant gingival tissues. Both rosiglitazone-induced glycemic control and glycemic fluctuation caused microbiota profile change in diabetic mice compared to that in uncontrolled hyperglycemic mice.

**Conclusions:**

This study suggests that glycemic fluctuation may aggravate peri-implantitis inflammation and bone loss, which may be associated with a shift in peri-implant microbial profile towards dysbiotic changes and the activation of TLR2/4-IRAK1-TRAF6 signaling.

**Supplementary Information:**

The online version contains supplementary material available at 10.1186/s40729-021-00360-9.

## Background

Type 2 diabetes mellitus (T2DM) has emerged as an increasingly common disease during last decades [1]. Substantial evidence in literature has well documented the positive correlation between T2DM and the prevalence and severity of periodontal disease [2], and it is more common for T2DM patients to have multiple lost teeth as a result of periodontal disease. Dental implant has become an indispensable treatment option in dentistry for the replacement of missing teeth [3]. Recent studies indicate that dental implant treatment can be safely carried out in diabetic patients with well-controlled blood glucose [4, 5]. However, it has been shown that maintenance of excellent glycemic stability is difficult to achieve in many diabetic patients, and hyperglycemia impairs bone healing and osseointegration around dental implants [6].

Peri-implantitis, a gingival inflammation which affects both soft and hard tissues surrounding the implant, has become one of the most common complications of dental implants. Poorly controlled T2DM patients exhibit more peri-implantitis and worse implant survival rate [6]. Although high blood glucose in T2DM is recognized to negatively impact alveolar bone repair and dental implant treatment [7], studies of the influences of metabolic changes or varying glucose levels on the process of osteoplastic matrix synthesis is unclear. Further analysis is required to determine the pathogenic roles of different glycemic conditions in periodontal bone loss and inflammatory response of experimental peri-implantitis.

TLR2/4 signaling pathway has been shown to play a key role in the initiation of inflammation processes in many diseases [8, 9]. Stimulation of TLR2/4 or IL-1R leads to activation of sequential downstream factors, including the IL-1 receptor-associated kinases (IRAKs) and TRAF6 via the adapter protein myeloid differentiation factor 88 (MyD88) [10]. This eventually activates the transcription factor NF-κB, which regulates a large range of cytokines, including IL-1β, TNF-α, and RANKL/OPG; many of them have been shown to play a pathologic role in peri-implantitis [11]. In our previous studies, we have demonstrated that the TLR2/4 signaling pathway is aberrantly upregulated in many tissues in diabetic animals, which has been associated with the increase of pro-inflammatory factors in these tissues [11–13]. Notably, the inhibition of the TLR2/4 signaling pathway decreases the levels of such inflammatory cytokines in diabetic animals, indicating a causative relationship between TLR2/4 and inflammation in diabetes [13].

In the present study, we determined the effects of changing glycemic conditions on TLR2/4 signaling and pro-inflammatory cytokines levels in peri-implant tissues, and on the subsequent bone resorption, using an experimental peri-implantitis diabetic murine model. Additionally, we evaluated the potential oral microbiota profile changes in these different glycemic conditions.

## Methods

### Materials

Rosiglitazone, sulfamethoxazole, trimethoprim, paraformaldehyde, phosphate buffer saline (PBS), ethylenediaminetetraacetic acid (EDTA), acid phosphatase, leukocyte (TRAP) kits and GenElute Bacterial DNA kits were purchased from Sigma-Aldrich Inc. (St. Louis, MO). MiR-146a mimic and anti-receptor activator of NF-κB ligand (RANKL) antibody (Ab) were from R&D Systems (Minneapolis, MN). Screw-shaped titanium implant was from D. P. Machining Inc. (Fenton, MI). TRIzol Reagent and 7-0 silk ligature were from Thermo Fisher Scientific (Waltham, MA). SuperScript First-Strand Synthesis system, SYBR Green I Master Mix, LightCycler 480 II real-time PCR system, and primers of tumor necrosis factor-α (TNFα), interleukin-1β (IL1β), IL10, IL-17, RANKL, osteoprotegerin (OPG), Toll-like receptor2 (TLR2), TLR4, interleukin-1 receptor-associated kinase 1(IRAK1), tumor necrosis factor receptor-associated factor 6 (TRAF6), nicotinamide phosphoribosyl transferase(NAMPT), silent mating type information regulation 2 homolog 1 (SIRT1), and β-actin were from Invitrogen (Carlsbad, CA).

### Animals

Wild-type (WT) C57BL/6 and leptin receptor-deficient (db/db) mice (4 weeks of age) were purchased from Jackson Laboratory (Bar Harbor, ME). Mice were fed a soft diet ad libitum for the duration of the experiment. All animal experiments were in accordance with the guidelines for treatment of animals in research outlined by the Institutional Animal Care and Use Committee of the Forsyth Institute (No. 17-022). WT mice were used as healthy implant control group. Db/db mice were randomly assigned to 5 groups: diabetic implant without glycemic control (DM), diabetic peri-implantitis without glycemic control (DM+lig), diabetic peri-implantitis with blood glucose control (DM+lig+BG control), diabetic peri-implantitis with poor glycemic control (DM+lig+BGswing), and diabetic peri-implantitis with peri-implant prevention without glycemic control (DM+lig+RANKL Ab+miR146a) (6 mice in each group).

### Tooth extraction, implantation, peri-implantitis induction, and peri-implant treatment

Left maxillary first and second molars were extracted in all mice, and the extraction sites were allowed to heal for 6 weeks. Mice were given sulfamethoxazole and trimethoprim (850 μg/170 μg/mL) in the drinking water for 2 weeks. Six weeks later, implants were placed in all animals as previously described [12, 13]. Briefly, gingival tissue corresponding to the extraction site was punched, and a 0.3-mm-diameter and 1.0-mm-depth hole was drilled in alveolar bone. A smooth-surface, screw-shaped titanium implant (1 mm in length and 0.5 mm in diameter) was screwed into the maxillary bone and allowed to heal for 4 weeks. During implant healing, the antibiotics and powder food were given to mice as described above. Four weeks after implant insertion, experimental peri-implantitis was induced by subgingival placement of a 7-0 silk ligature around each implant immediately apical to the implant head in DM+lig, DM+lig+BG control, DM+lig+BG swing, and DM+lig+RANKL Ab+miR146a groups. Upon ligation, weekly injection of 0.1 mL mixture (containing 2.5 mg/kg bodyweight of RANKL Ab and 1.5 μg of miR-146a mimic) were used in the mesial and distal gingival papilla, respectively, in DM+lig+RANKL Ab+miR146a group. There two agents have been used in the previous studies for the inhibition of local periodontal inflammation and bone loss [10, 14]. All ligatures were removed after 3 weeks. The entire experimental process was described in supplemental figure S1. Tooth extraction, implantation, ligation, peri-implant injection, and ligature removal were performed under general anesthesia by intraperitoneal administration of ketamin (100 mg/kg) and xylazine (5 mg/kg). At the end of the experiment (4 weeks after the ligature placement, week 14 in the experiment), mice were euthanized by the CO_2_ inhalation method.

### Bodyweight measurement, blood glucose detection, and glycemic control

Db/db mice in DM, DM+lig, and DM+lig+RANKL Ab+miR146a groups consumed a standard semi-synthetic diet (AIN-93G) (standard diet) during the experiment. Animals in the other 2 diabetic groups also consumed standard diet before implant surgery; however, after surgery, mice in DM+lig+BG control group consumed AIN-93G diet supplemented with rosiglitazone (0.005% w/w) (rosiglitazone diet) every day, and those in DM+lig+BG swing group consumed rosiglitazone diet for 1 week in every other week (1-week rosiglitazone diet followed by1-week standard diet in each cycle). Bodyweight and blood glucose tests were carried out from 4 weeks of age (week 0 in the experiment) weekly. Glucose in blood collected from tails was monitored using OneTouchGlucometer (LifeScan, Milpitas, CA), and fasting blood glucose (FBG) levels were determined following a 12 h fast. Fasting glucose over 12 mmol/l was considered diabetic [15]. In this study, 10 μL blood from the tail vein of each mouse were used for fasting blood glucose test at a time. The test was carried out three times and the average was used for each animal.

### Micro-computed tomography scanning

At sacrifice, all mouse maxillae were fixed in 4% paraformaldehyde for examination using micro-computerized tomography (micro-CT) (mCT-40, Scanco Medical) (gingival tissues around each implant were collected from 3 maxillae in each group before paraformaldehyde treatment). All samples were exposed to polychromatic X-rays on a rotating stage at a steep angle of 0.18° over 360°, and measured at an operating voltage of 70 kVp and 114 mA current and 6 mm isotropic voxel resolution. Afterwards, volumetric data were converted to DICOM format and used to generate reconstructed images using software Amira (FEI Visualization Sciences Group). To quantify the alveolar bone loss around the implant, volume of interest (VOI) was defined by a cylinder with a diameter of 1.0 mm and a height of 1.0 mm from the top surface of each implant. The bone loss surrounding implants was calculated by total VOI volume (TV) minus total bone volume (BV) in 3D morphometric analysis [11].

### Hematoxylin and eosin staining

The scanned maxillae with gingival tissues (3 samples per group) were decalcified with 10% EDTA solution. Decalcification continued for 3 weeks at 4 °C, and the decalcification solution was changed every other day. Fully decalcified samples were embedded in paraffin and cut into sections at the mesiodistal plane for hematoxylin and eosin (H&E) staining as previously reported [11]. Stained sections were photographed under a light microscope (Olympus Corp., Tokyo, Japan). At an objective magnification of × 40, numbers of inflammatory cells in 4-unit squares (50 μm × 50 μm) in the connective tissue surrounding each implant were counted, and then averaged to represent the inflammatory infiltrate level per mouse.

### Tartrate-resistant acid phosphatase (TRAP) staining

The scanned specimens with gingival tissues (3 samples per group) were decalcified in 10% EDTA solution, embedded, and sectioned at the mesiodistal plane. The resulting sections were stained using TRAP Kits, as previously described [16]. Images of gingival areas mesial and distal to the implants from each section were acquired under a light microscope (Olympus Corp., Tokyo, Japan) at an objective magnification of × 40. The number of multi-nucleated TRAP positive cells along alveolar bone surface was counted for each sample and averaged per group. Averaged TRAP positive cell numbers were normalized to the wild-type group to demonstrate fold difference.

### Real-time quantitative RT-PCR

Gingival tissues immediately adjacent to the implant (3 samples per group) were harvested and treated with TRIzol Reagent (Thermo Fisher Scientific, Waltham, MA), according to the manufacturer’s protocol. cDNA was synthesized from total RNA extracted using the SuperScript First-Strand Synthesis system and amplified using SYBR Green I Master Mix with the LightCycler 480 II real-time PCR system with primers. Amplified β-actin gene was used as an internal control. The primer sequences used in this study were in Supplemental Table 1.

### Microbial16S rRNA sequencing analyses

Saliva samples from oral cavity (3 samples per group) were collected using rayon swabs and placed in 200 μL PBS at week 6 and week 14 in the experiment (week 6, collected before implant insertion and glycemic control, and week 14, collected before sacrifice). Ligature samples (3 samples per group) were collected at week 14 before sacrifice. Then, the bacteria in the samples were identified by full-length 16S ribosomal RNA gene sequencing as reported previously [17]. Whole genomic DNA was extracted from the swabs using the GenElute Bacterial DNA Kit and used as templates in 16S ribosomal RNA (rRNA) gene next-generation sequencing analyses following the manufacturer’s instructions. The sequence read pairs were merged to single reads with a script (join_paired_ends.py) provided by the Quantitative Insights into Microbial Ecology (QIIME) package (v1.91) [17].

### Statistics

Data are expressed as mean ± SD. Differences in parameter mean values were analyzed using one-way analysis of variance (ANOVA) test followed by SNK-*q* multiple comparisons using GraphPad 6.0 Software (La Jolla, CA). A *P*-value of < 0.05 was considered statistically significant.

## Results

### Glycemic control does not affect bodyweights but blood glucose of diabetic mice

As shown in supplemental Fig. S2A, bodyweights of all diabetic mice increased steadily, and faster than WT mice during the experiment. Additionally, rosiglitazone treatment did not have effects on the bodyweight of diabetic mice. As observed in supplemental Fig. S2B, fasting blood glucose (FBG) levels in diabetic mice with daily rosiglitazone treatment were effectively controlled to normal level, while irregular rosiglitazone treatment cycle induces blood glucose fluctuation in diabetic animals. The mice in DM+lig+BG swing group exhibited excellent glycemic control after the week of rosiglitazone addition but high glucose levels after the week without rosiglitazone treatment in each cycle. After implant insertion surgery, FBG levels remained high in all diabetic mice without rosiglitazone application.

### Glycemic fluctuation elevated peri-implant bone loss in diabetic mice

**Fig. 1:**
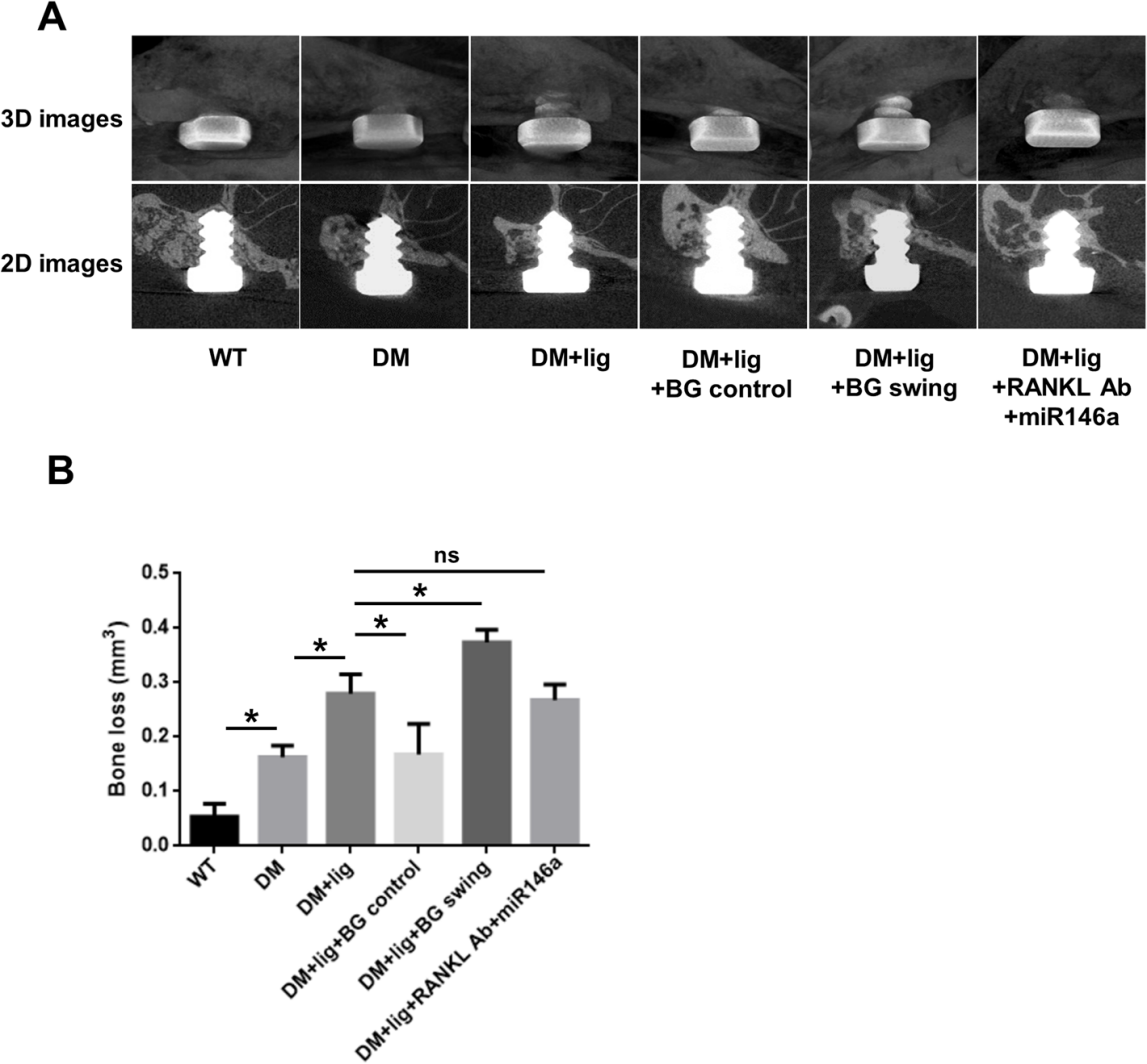
Peri-implant bone loss detected using micro-CT. **a** Micro-CT images of mesial-distal bone surrounding implants in different groups of mice. **b** The peri-implant bone loss was calculated in 3D morphometric analysis (mean ± SD, n = 6, *p < 0.05; ns, no significance

Bone loss around the implants was significantly increased in all diabetic groups, compared with WT group (Fig. 1a, b). Moreover, ligation enhanced peri-implant bone loss levels in diabetic mice (DM+lig vs. DM), and glucose fluctuation further elevated the bone loss level (DM+lig swing vs. DM+lig) (Fig. 1b). Upon excellent glycemic control, bone loss was reduced in diabetic mice with peri-implantitis (DM+lig+BG control vs. DM+lig), whereas local injection with anti-RANKL antibody and miR146a showed no obvious effects on bone loss in peri-implantitis with uncontrolled diabetes (DM+lig+RANKL Ab+miR146a vs. DM+lig).

### Glycemic fluctuation aggravates peri-implant inflammatory cell infiltration in diabetic mice

**Fig. 2:**
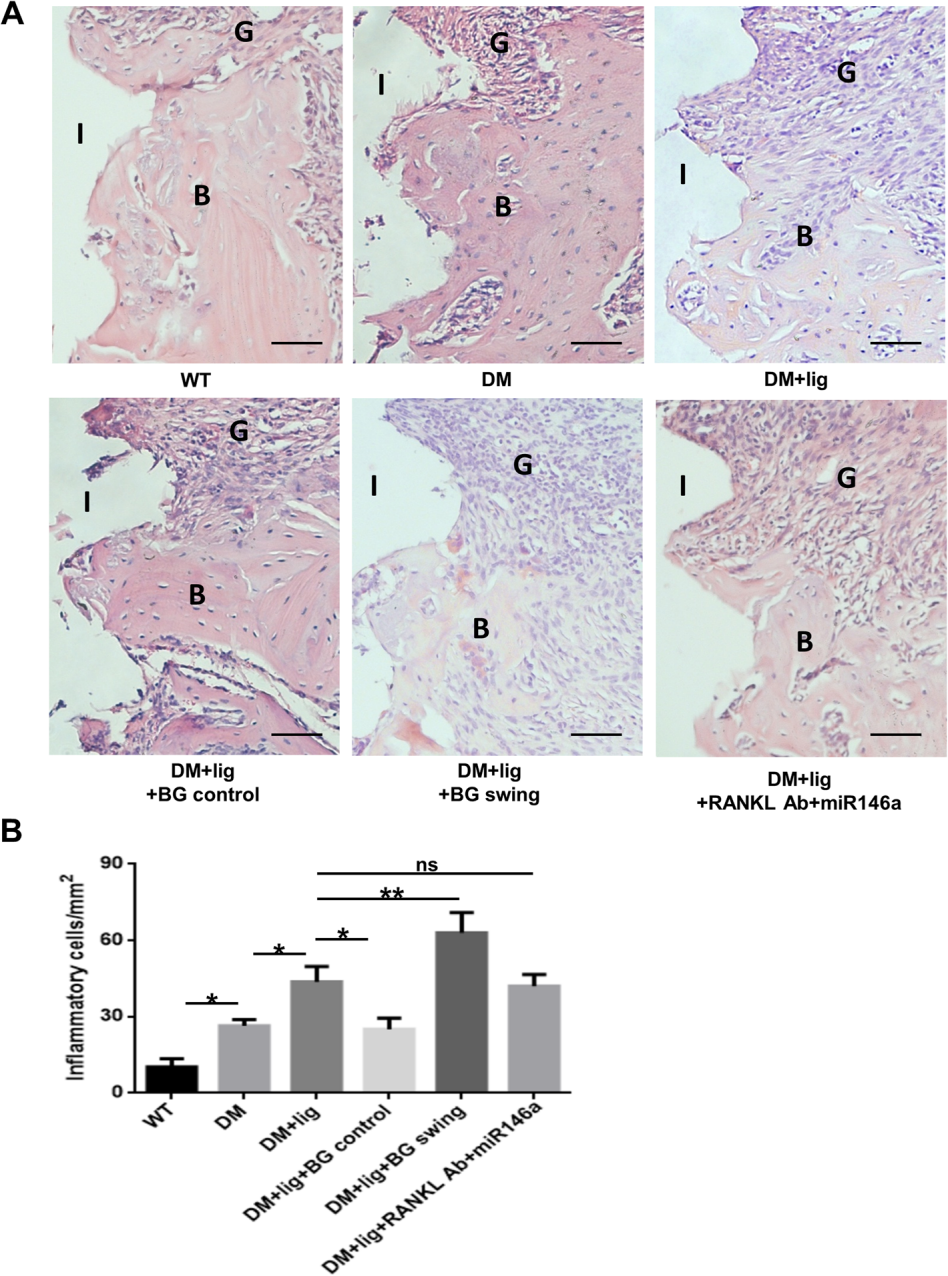
Inflammatory infiltrate in peri-implant tissues was examined using H&E staining. **A** Images of tissues surrounding implants in different groups of mice. **B** Numbers of infiltrated inflammatory cells in peri-implant tissues were analyzed (mean ± SD, n = 3, *p < 0.05, **p < 0.01; ns, no significance; I, implant space; B, bone; G, gingiva. Scale bars, 50 μm)

Similar to the changes in bone loss level, peri-implant inflammatory cell infiltration was greater in DM mice than in WT mice and in DM+lig mice than in DM mice (Fig. 2A, B). Glycemic control decreased inflammatory infiltrate in peri-implant connective tissues (DM+lig+BG control vs. DM+lig), while the inflammatory infiltrate was further increased in diabetic mice with blood glucose fluctuation as compared to mice with sustained hyperglycemic level (DM+lig+BGswing vs. DM+lig) (Fig. 2B). Local anti-osteolytic/anti-inflammatory treatment showed no significant effects on peri-implant inflammatory infiltrate in peri-implantitis under diabetic conditions (DM+lig+RANKL Ab+miR146a vs. DM+lig) (Fig. 2B).

### Glycemic fluctuation exacerbates peri-implant osteoclast formation in diabetic mice

**Fig. 3:**
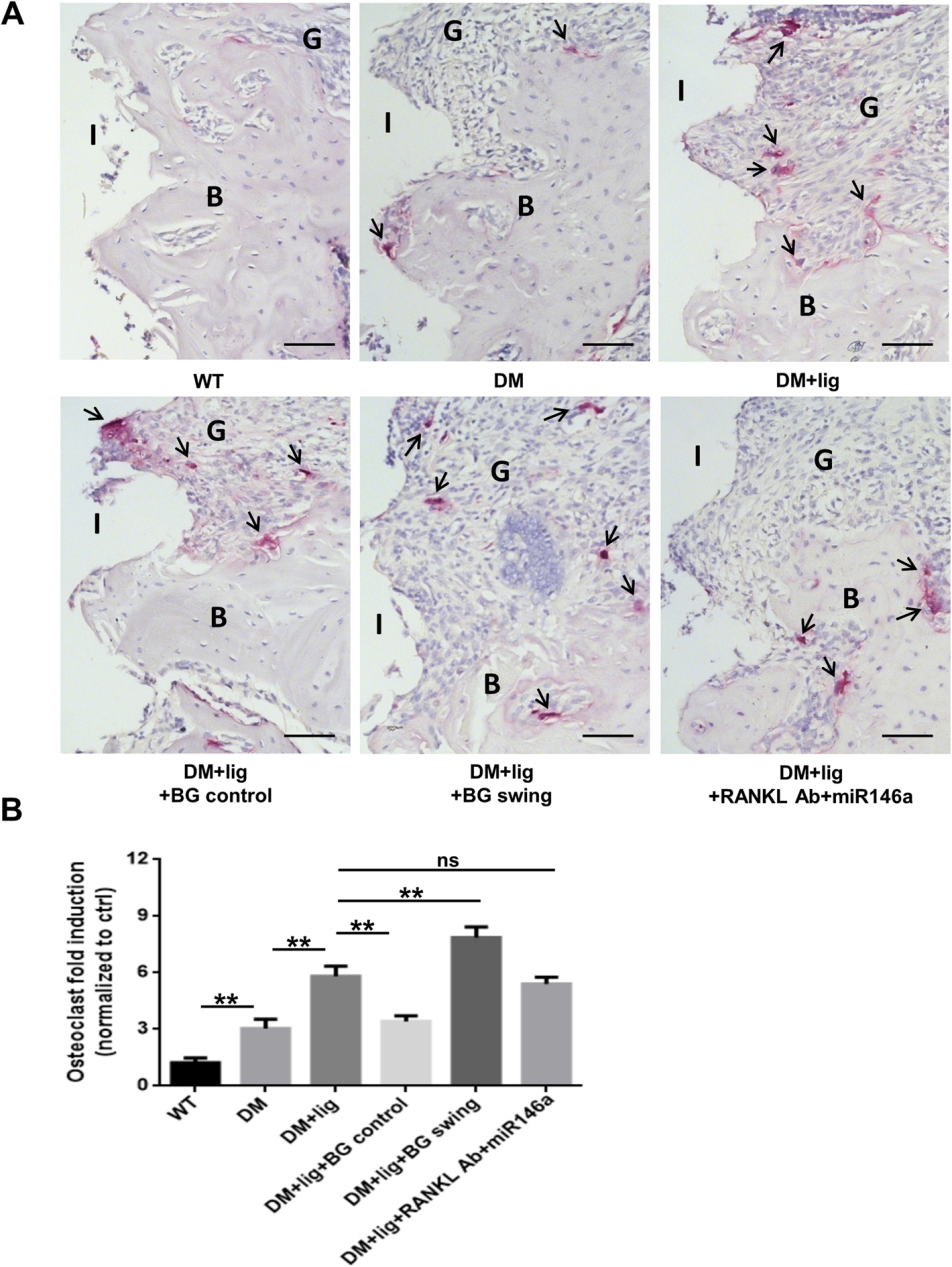
Osteoclast formation in peri-implant tissues was detected using TRAP staining. **A** Histological images of TRAP staining of peri-implant tissues of different groups of mice. (black arrows, TRAP positive osteoclasts). **B** Numbers of osteoclasts in peri-implant tissues were analyzed (n = 3) (mean ± SD, n = 3, *p < 0.05, **p < 0.01; ns, no significance; I, implant space; B, bone; G, gingiva. Scale bars, 50 μm)

Peri-implant osteoclast formation was assessed by TRAP staining (Fig. 3A). Results showed an increase in TRAP positive cell formation in DM peri-implant tissues, compared with WT peri-implant tissues (Fig. 3B). The level of TRAP positive cell formation was further elevated in DM+lig group, compared with DM group. Upon regular glycemic control, this level was downregulated dramatically (DM+lig+BG control vs. DM+lig) tissues (Fig. 3B). However, the level was significantly upregulated under fluctuated hyperglycemic condition (DM+lig+BG swing vs. DM+lig). There was no significant difference in osteoclast formation between DM+lig+RANKL Ab+miR146a group and DM+lig group (Fig. 3B), indicating peri-implant local treatment alone could not ameliorate increased osteoclast formation in peri-implant tissues under diabetic condition.

### Glycemic fluctuation alters gingival mRNA expressions of inflammatory proteins in diabetic mice

**Fig. 4:**
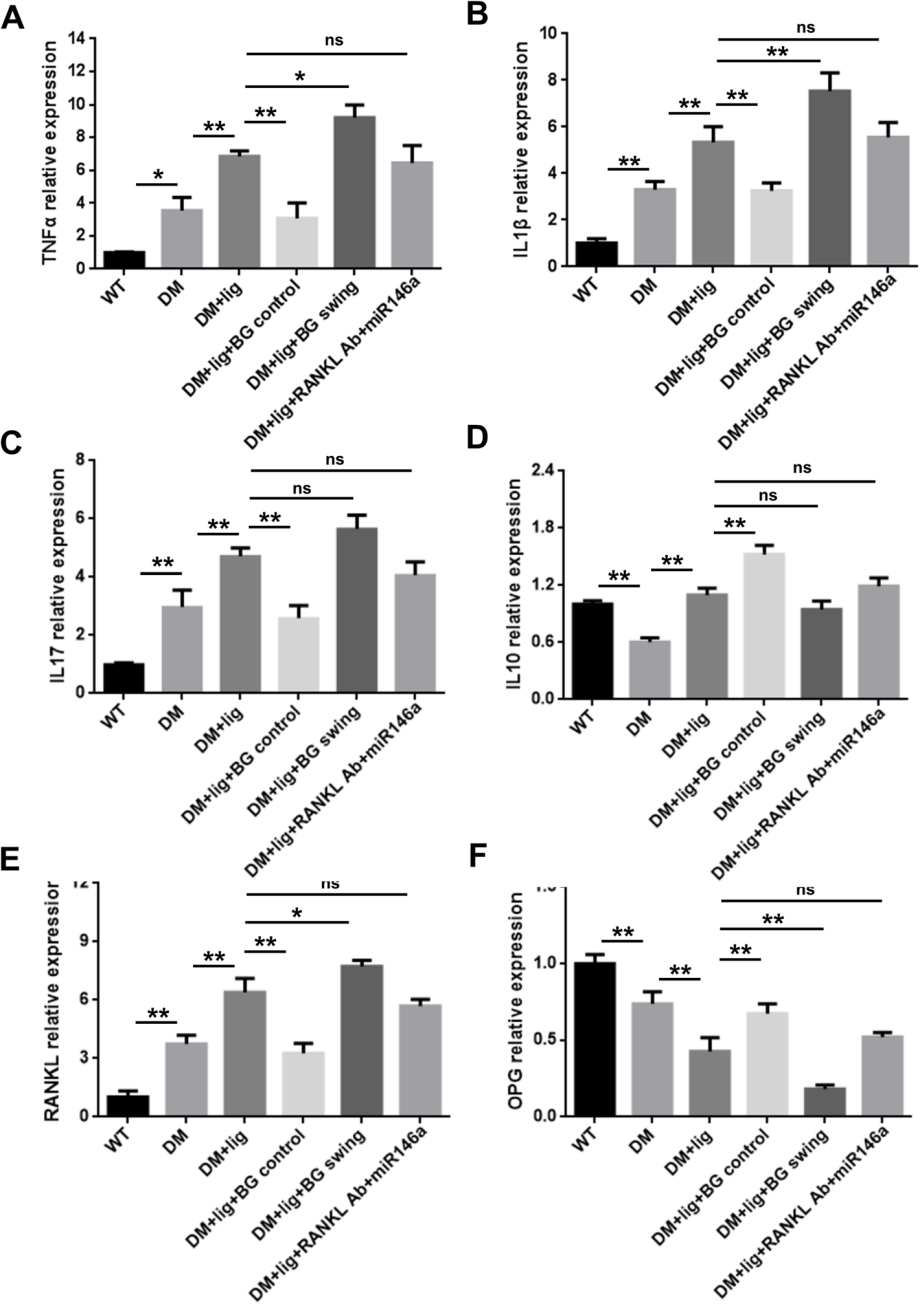
The mRNA expression of inflammatory cytokines and bone formation proteins in peri-implant gingival tissues. Gingival tissues around ligatured site were excised and processed for RT-qPCR analyses to determine mRNA level of **a** TNF-α, **b** IL-1β, **c** IL-17, **d** IL-10, **e** RANKL, and **f** OPG (mean ± SD, n = 3, *p < 0.05, **p < 0.01, ns, no significance)

Diabetes increased the mRNA expression of TNFα (Fig. 4a), IL1β (Fig. 4b), and IL-17 (Fig. 4c) in peri-implant gingival tissues (DM vs. WT), and peri-implantitis further upregulated the expression levels of these pro-inflammatory proteins (DM+lig vs. DM). Additionally, glycemic control decreased these mRNA expressions (DM+lig+BG control vs. DM+lig). Glycemic fluctuation (DM+lig swing vs. DM+lig) further enhanced all the mRNA expression levels except IL-17 expression. Moreover, local anti-osteolytic/anti-inflammatory treatment did not alter the mRNA expressions of these cytokines in diabetes with peri-implantitis (DM+lig+RANKL Ab+miR146a vs. DM+lig). A reduced level of IL10 mRNA expression was observed in DM mice compared to WT mice. However, induction of peri-implantitis elevated the expression level of IL10 (DM+lig vs. DM), whereas glycemic control markedly enhanced such increase of IL10 mRNA expression (DM+lig+BG control vs. DM+lig) (Fig. 4d). DM+lig swing and DM+lig+RANKL Ab+miR146a mice exhibited similar IL10 mRNA expression levels to DM+lig mice (Fig. 4d). The alteration of RANKL mRNA expression was in line with the TNFα mRNA, and OPG mRNA expression was reversely regulated relative to RANKL mRNA expressions (Fig. 4e, f). Local injection of anti-RANKL antibody and miR146a exhibited no significant effect on RANKL and OPG mRNA expression (Fig. 4e, f).

### Differential expressions of TLR signaling molecules in diabetic mice

**Fig. 5:**
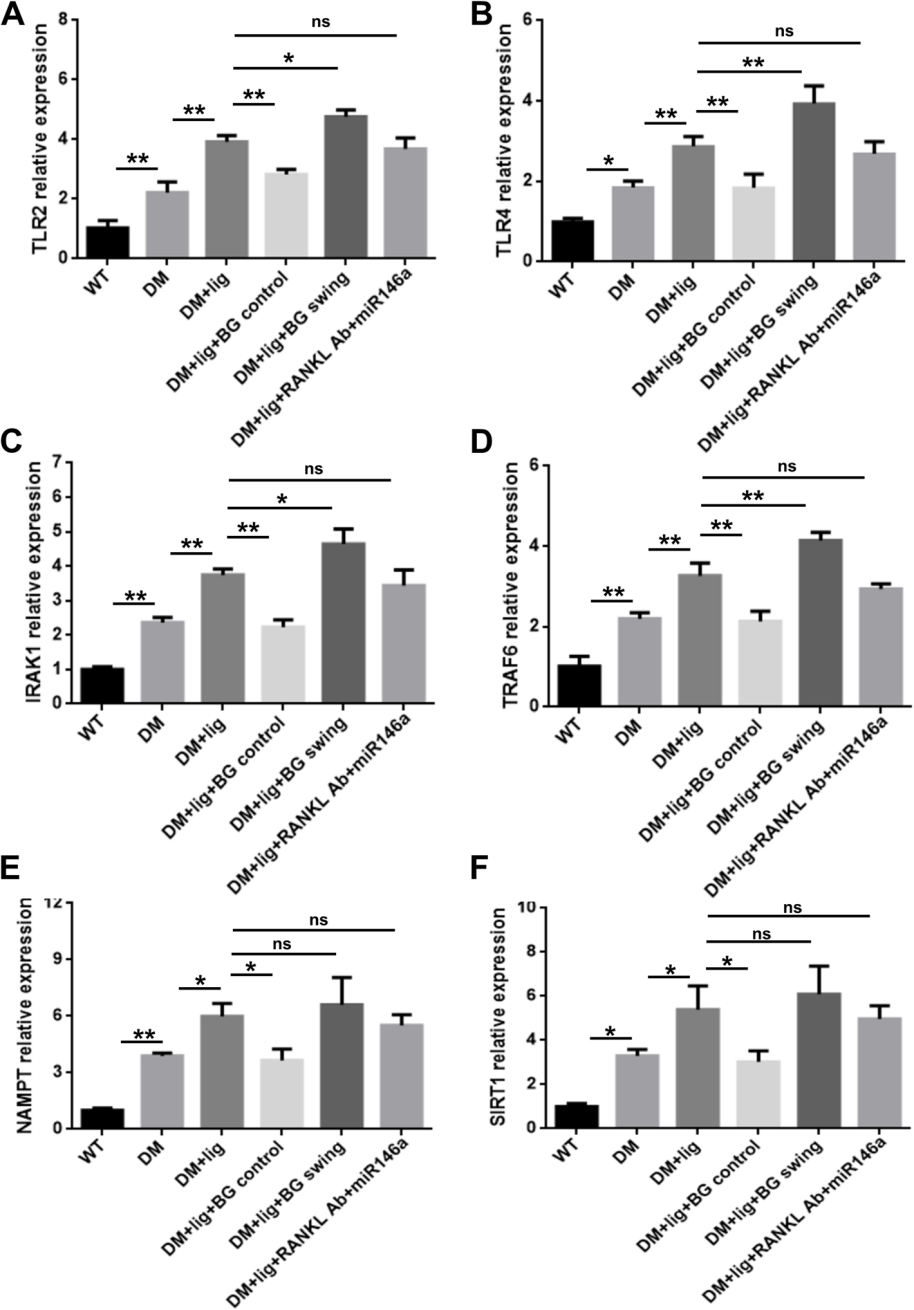
The mRNA expression of TLR and NAMPT signaling members in peri-implant gingival tissues. Gingival tissues around ligatured site were excised and processed for RT-qPCR analyses to determine mRNA level of **a** TLR2, **b** TLR4, **c** IRAK1, **d** TRAF6, **e** NAMPT, and **f** SIRT1 (mean ± SD, n = 3, *p < 0.05, **p < 0.01, ns, no significance)

TLR2/4mRNA expressions were significantly higher in DM group than in WT group and in DM+lig group than in DM group (Fig. 5a, b). In addition, compared with DM+lig group, DM+lig+BG control group had lower TLR2/4 expressions, whereas DM+lig swing group had higher TLR2/4 expressions (Fig. 5a, b). No significant difference was observed in TLR2/4 expressions between DM+lig and DM+lig+RANKL Ab+miR146a groups (Fig. 5a, b). The alteration of IRAK1 (Fig. 5c) and TRAF6 (Fig. 5d) mRNA expressions were similar to the TLR2/4 mRNA expressions. Significantly lower NAMPT (Fig. 5e) and SIRT1 (Fig. 5f) expressions of mRNA levels was shown in WT mice than in DM mice and in DM mice than in DM+lig mice. Upon glycemic control, NAMPT and SIRT1 mRNA expressions were repressed (DM+lig+BG control vs. DM+lig). No significant effects on NAMPT or SIRT1 mRNA expression were observed in DM+lig swing orDM+lig+RANKL Ab+miR146a prevention group when compared with DM+lig group (Fig. 5e, f).

### Manipulation of glycemic levels altered profiles in salivary and peri-implant microbiota of diabetic mice

**Fig. 6:**
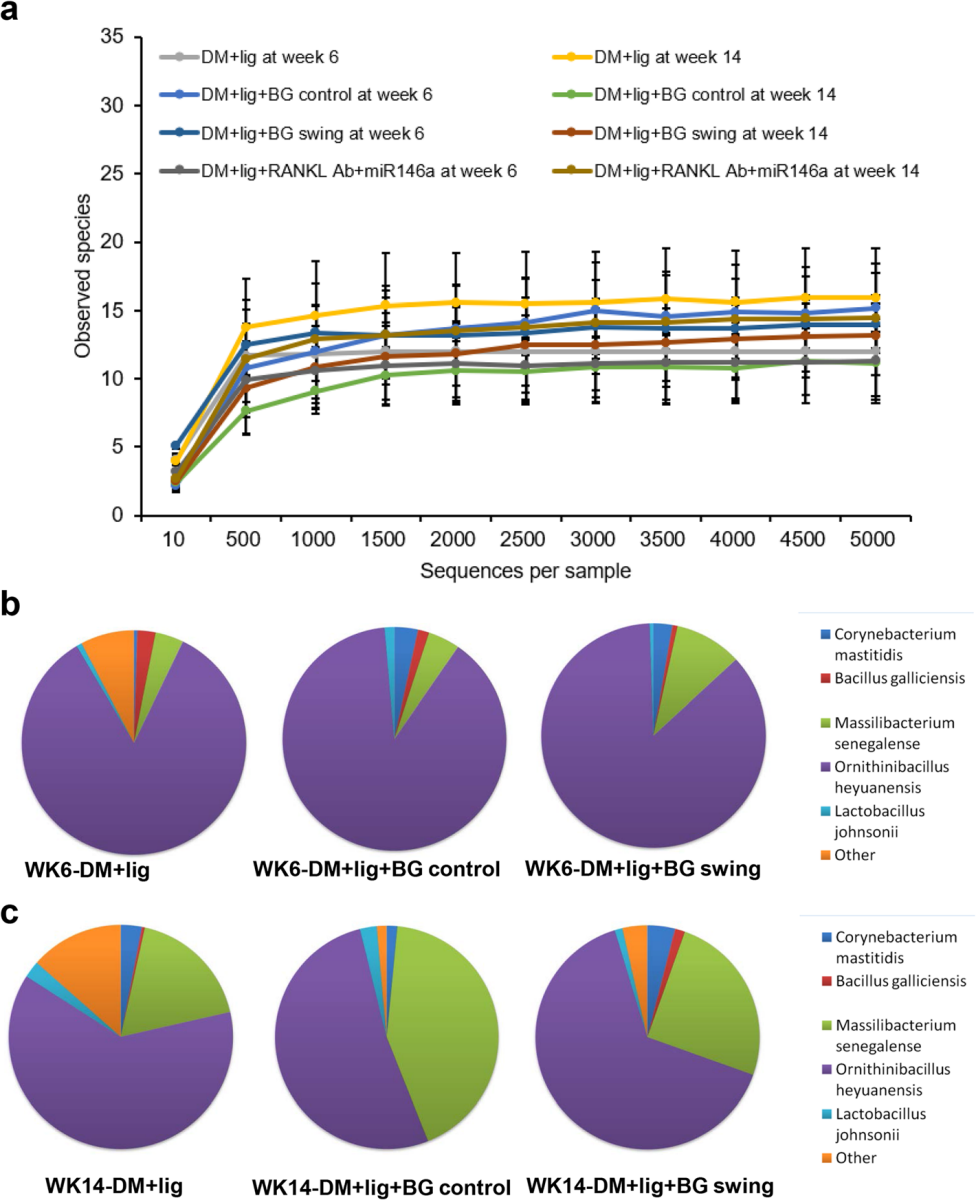
Species of saliva oral bacteria were analyzed using 16S rRNA gene sequence. **a** Total numbers of observed species of oral bacteria from different groups at all timepoints (mean ± SD, n = 3). **b** Distribution of observed species of oral bacteria at WK6 (week 6), saliva samples from oral cavity were collected before implant insertion and glycemic alteration. **c** Distribution of observed species of oral bacteria at WK14 (week 14), saliva samples from oral cavity were collected before sacrifice

To detect whether glycemic condition affects the oral microbiota profile, we analyzed the 16S rRNA gene sequence of the saliva bacterial samples from mouse oral cavities at week 6 and week 14 in the experiment. Our results showed no significant differences among total numbers of observed species of oral bacteria from different groups at each timepoint, and from the same group at different timepoints (Fig. 6a). However, when the composition of the same diabetic group at different timepoints was compared, they exhibited different bacterial communities. From week 6 to week 14, the species of the bacterial population were retained, but the abundance of *Massilibacterium senegalense* increased, and *Ornithinibacillus heyuanensis* became less dominant (Fig. 6b, c). The bacterial composition of healthy controls did not change over time and was similar to those diabetic groups at week 6 (data not shown). This transmission of oral microbial populations was not associated with glycemic control or peri-implantitis severity, but correlated with the duration of diabetes (Fig. 6b, c). Moreover, the bacterial samples isolated from ligatures at week 14 were analyzed for diversity and composition of bacterial communities. The Chao1 Index demonstrated that there are similar numbers of species in the DM+lig group and DM+lig+BG swing group, but a reduced number of species in the DM+lig+BG control group (supplemental Fig. S3A). Consistently, the heatmap of species abundance showed the similar results indicating a closer microbial profile between the DM+lig group and DM+lig+BG swing group as compared to the DM+lig group (supplemental Fig. S3B). However, species abundance variations were also detected when comparing DM+lig+BG swing group vs. DM+lig group, or DM+lig+BG control group vs. DM+lig group (supplemental Fig. S3B), likely associated with the glycemic fluctuation or glycemic control, respectively.

## Discussion

A systemic review of diabetic patients has shown the success rate of dental implants determined largely by glucose control [18]. Poorly controlled diabetic patients often exhibit a greater tendency of peri-implantitis and higher failure rate of implant therapy [6]. Several authors reported that patients with diabetes are at higher risk for peri-implantitis [19, 20]. However, these reports were not adjusted for potential confounding, and diabetes as a potential risk factor for peri-implantitis may be inconclusive [21]. In a retrospective study, diabetic patients with mucositis were not at higher risk to develop peri-implantitis when compared to non-diabetics [22]. Different cross-sectional studies also showed a lack of association between peri-implantitis and diabetes [23–25]. Both high blood glucose and glucose fluctuation in diabetes impair alveolar bone defect healing [26, 27], while it remains unclear in previous studies which status has more adverse effects in dental implant treatment. In this study, uncontrolled hyperglycemic status increased peri-implant bone loss in peri-implantitis, while glucose fluctuation further enhanced the bone loss level, indicating that compared with sustained hyperglycemia, glycemic fluctuation may have worse impacts on peri-implantitis osteolysis. Additionally, excellent glycemic control reduced the bone loss around implants, but local anti-osteolytic/anti-inflammatory treatment showed no significant protective effect, suggesting greater impact of systemic hyperglycemia on peri-implantitis pathogenesis, and further advocates the importance of glycemic control in peri-implantitis with diabetes.

In this study, after implant surgery, mice in DM+lig+BG control group consumed diet with rosiglitazone every day, and those in DM+lig+BG swing group consumed rosiglitazone diet for 1 week in every other week. The change in rosiglitazone administration could contribute to blood glucose fluctuation and may or may not lead to variation in insulin secretion and HbA1c levels. However, this study focused on the association between glycemic fluctuation and change in peri-implant tissue inflammation and bone loss; therefore, we tested blood glucose only. Future studies are warranted to determine the effect of insulin and HbA1c variation on peri-implant inflammation and bone loss.

Inflammatory infiltrate and osteoclast formation in the diseased lesions are considered two important parameters to represent the severity of peri-implantitis [28]. In the present work, the change in these two parameters was in accordance with that in bone loss in each group, suggesting greater inflammatory response in peri-implant tissues caused by glycemic fluctuation, compared with persistent hyperglycemia. Moreover, the observation indicates amelioration of peri-implantitis by good glucose control but not local anti-osteolytic/anti-inflammatory treatment alone. Animal research has shown that chronic hyperglycemia increases inflammatory infiltrate in periodontal inflamed lesions, which leads to higher levels of pro-inflammatory cytokines and tissue damage [29, 30]. Long-lasting hyperglycemia also promotes the proliferation and differentiation of osteoclasts, resulting in bone tissue more susceptible to resorption [31, 32]. RANKL Ab and miR146a have been proven to negatively modulate inflammatory responses in the periodontium or periodontal cells [33, 34]. Local RANKL Ab or miR146a administration can inhibit osteoclast formation and reduce bone destruction in inflammatory diseases [34, 35]. However, such treatments alone may not be sufficient to overcome the impact of systemic inflammation under diabetic condition. Both glycemic control and local treatments in oral cavity are critical for diabetic patients who exhibit implant survival similar to that in systemically healthy individuals [36]. However, to date, there is no report on the comparison for the treatment effect of glycemic control and oral measures in the maintenance of dental implants in diabetic patients. Future studies are warranted to determine the correlations and efficacy of such combination of systemic and local treatments.

To further investigate the underlying mechanism of the impact of glycemic alteration on peri-implantitis, we detected the change of TLR signaling and downstream mediators in peri-implant gingival tissues. Our data showed that the levels of TLR2, TLR4, IRAK1, and TRAF6 gene expression were in agreement with that in bone loss, suggesting peri-implantitis augmented by glycemic fluctuation were at least partly mediated through the activation of TLR2/4 signaling (Fig. 5). Additionally, our results indicate that excellent glycemic control might attenuate peri-implantitis through inhibiting TLR2/4 signaling. The activation of TLR2/4 signaling is positively associated with the progression of T2DM and periodontitis [37, 38]. TLR2 and TLR4 are implicated in the excessive inflammation under diabetic conditions [39]. They can activate their downstream adaptors IRAK1 and TRAF6 and subsequently induce the secretion of cytokines, such as TNFα, leading to the aggravation of inflammatory responses [39].

Rosiglitazone is a synthetic PPARγ agonist and lowers blood glucose through enhancement of the insulin sensitivity of the target cell [40]. Interestingly, in the bone, PPARγ activation and insulin play opposing roles in the process of bone formation. Insulin is able to not only stimulate glucose update directly via binding to receptors on osteoblasts, but also can enhance bone formation through enhancement matrix synthesis and interference with the production of a mineralized matrix [41, 42]. Unlike the restorative effects of insulin, activation of PPARγ suppresses osteoblasogenesis and activates osteoclastogenesis [43, 44]. In our study, we observed ligation enhanced peri-implant bone loss in diabetic mice but not in non-diabetic mice or in diabetic mice with rosiglitazone treatment, indicating that hyperglycemia has an adverse effect on bone metabolism. When treating diabetic mice with interrupted rosiglitazone, blood glucose levels fluctuated, indicating an insufficient level of insulin activity. Furthermore, bone loss in peri-implant tissues of these mice was remarkably higher than those in diabetic mice with normoglycemia or persistent hyperglycemia, suggesting that ineffective insulin signaling may aggravate the process of bone loss. One noteworthy finding is that there is no significant difference between the amount of bone loss in non-diabetic mice and that in normoglycemic diabetic mice treated with rosiglitazone, suggesting that the adverse effect of PPARγ activation on bone is negligible in peri-implant tissue. Further studies need to address the detailed mechanism of PPARγ activation on bone loss of peri-implantitis.

Although augmented host immune response is critical in alveolar bone loss in diabetes [45], the role of alteration in oral microbiota should not be neglected. A recent research has shown changes in oral microbial diversity and an increase in alveolar bone loss in diabetic mice [17]. The present study showed that the diversity of oral salivary bacteria was similar in different groups with certain composition changes from initial stage to the end stage of the experiment (Fig. 6). The abundance of *Massilibacterium senegalense* increased and that of *Ornithinibacillus heyuanensis* decreased in all diabetic groups over time. These observations also suggest the dysbiotic bacterial communities in diabetes. This alteration may change the pathogenicity of oral microbiota in diabetes compared with healthy status. *Massilibacterium senegalense* is a Gram-negative bacterium [46], and *Ornithinibacillus heyuanensis* a Gram-positive bacterium [47]. Although the impact of these two species on periodontitis or peri-implantitis has not been confirmed, it has been observed that a decrease in Gram-negative with an increase in Gram-positive bacteria in oral microbiota is accompanied with reduced inflammation in periodontitis [48]. Glycemic status in diabetes, such as continuous hyperglycemia and glucose fluctuation, can contribute to different inflammatory status. This may explain different alveolar bone loss levels with similar oral microbiota profile in different diabetic groups at each time point. Furthermore, peri-implant microbiota also showed variation in species when compare among uncontrolled hyperglycemia (DM+lig), glycemic fluctuation (DM+lig+BG swing) and glycemic control (DM+lig+BG control) groups (supplemental Fig. S3). These results suggested that manipulation glycemic levels in diabetes could alter the peri-implant microbial profile and potentially impact the outcomes of peri-implant inflammation and bone metabolism.

Oral microbiome has developed by modulating or avoiding inflammatory responses, and oral bacterial flora change in different diseases, including periodontitis [49]. TLR signaling pathways plays an important role in regulating host-microbe interactions, and oral epithelial cells respond to most periodontopathic bacteria via TLR2/4, leading to subsequent immune response in the periodontium [50]. However, the effect of TLRs on oral microbiota remains controversial. An experiment on mice demonstrated lack of TLR2 had a negligible effect on oral bacterial flora [51], whereas another report showed a significant change with a dominance of gram-negative species in the oral microbial composition in both TLR2 and TLR4 knockout mice, compared to the wild-type mice [49]. In the present study, the shift in oral microbial composition was only observed in diabetic groups and was irrelevant to the activation of TLR signaling. These results indicated that TLR2/4 signaling may not be associated with the changes in peri-implant microbiota under diabetic conditions. Further studies are needed to verify the role of TLR2/4 pathway in the regulation of peri-implant microbiota.

## Conclusion

In conclusion, our study suggests that glycemic fluctuation in diabetes causes more damage in peri-implantitis compared with uncontrolled hyperglycemia, through regulating TLR2/4-IRAK1-TRAF6 pathway but not NAMPT/SIRT1 signaling. Moreover, glycemic control but not local treatment alone can ameliorate peri-implantitis under diabetic conditions. These findings reiterated that glucose control is crucial to control peri-implantitis progression in diabetes. Furthermore, a profile change in salivary and peri-implant bacterial composition and abundance occurs in diabetes, implicating that specific bacterial species may serve as indicators for the development of peri-implantitis in diabetes.

## Supplementary Information

**Additional file 1: Table 1** List of primers used for real time quantitative RT-PCR. Supplemental FigureS1.Procedures of implantation, ligation, blood glucose control and treatment.1, Maxillary first and second molars were extracted; 2. Six weeks post-extraction, the implant was placed; Rosiglitazoneadministration was used to control blood glucose level.3. Four weeks after implant placement, the silk ligature was placed around implant; Gingival RANKL Ab and miR146a injection was performed. 4. Four weeks after ligation, mice were euthanized and samples were collected. Supplemental FigureS3.Species of ligation silk bacteria were analyzed using 16S rRNA gene sequence. The bacterial composition from ligation silks of different groups of mice was analyzed using 16S rRNA gene sequence. (A) Chao1 index of bacterialcommunity from ligation silks of DM+lig, DM+lig+BG control and DM+lig+BG swing groups (Mean ± SD, n = 3); (B) The heatmap of species abundance in DM+lig, DM+lig+BG control and DM+lig+BG swing groups.

## Data Availability

Presented in the main paper

## Abbreviations

PBS: Phosphate buffer saline
IL: Interleukin
TNFα: Tumor necrosis factor alpha
RANKL: Receptor activator of nuclear factor kappa-Β ligand
TLR: Toll-like receptor
IRAK1: IL-1 receptor-associated kinase-1
TRAF6: TNF receptor-associated factor 6
