# Supplementary material for: Glycemic fluctuation exacerbates inflammation and bone loss and alters microbiota profile around implants in diabetic mice with experimental peri-implantitis
Source: Int J Implant Dent. 2021 Aug 17;7:79. doi: 10.1186/s40729-021-00360-9 (PMC8368769; doi:10.1186/s40729-021-00360-9)
Supplement: Supplementary file 1 — Additional file 1: Table 1 List of primers used for real time quantitative RT-PCR. Supplemental FigureS1.Procedures of implantation, ligation, blood glucose control and treatment.1, Maxillary first and second molars were extracted; 2. Six weeks post-extraction, the implant was placed; Rosiglitazoneadministration was used to control blood glucose level.3. Four weeks after implant placement, the silk ligature was placed around implant; Gingival RANKL Ab and miR146a injection was performed. 4. Four weeks after ligation, mice were euthanized and samples were collected. Supplemental FigureS3.Species of ligation silk bacteria were analyzed using 16S rRNA gene sequence. The bacterial composition from ligation silks of different groups of mice was analyzed using 16S rRNA gene sequence. (A) Chao1 index of bacterialcommunity from ligation silks of DM+lig, DM+lig+BG control and DM+lig+BG swing groups (Mean ± SD, n = 3); (B) The heatmap of species abundance in DM+lig, DM+lig+BG control and DM+lig+BG swing groups. [file 40729_2021_360_MOESM1_ESM.docx]

**Table 1 List of primers used for real time quantitative RT-PCR**

| Target gene | Primer sequence |
| --- | --- |
| TNFα | Forward: 5’-CAACGCCCTCCTGGCCAACG-3’ |
|  | Reverse: 5’-TCGGGGCAGCCTTGTCCCTT-3’ |
| IL1β | Forward: 5’-ATGCCTTCCCCAGGGCATGT-3’ |
|  | Reverse: 5’-CTGAGCGACCTGTCTTGGCCG-3’ |
| IL10 | Forward: 5’-GACCAGCTGGACAACATACTGCTAA-3’ |
|  | Reverse: 5’-GATAAGGCTTGGCAACCCAAGTAA-3’ |
| IL-17 | Forward: 5’-CTCCAGAAGGCCCTCAGACTAC-3’ |
|  | Reverse: 5’-GGGTCTTCATTGCGGTGG-3’ |
| RANKL | Forward: 5’-CATGTGCCACTGAGAACCTTGAA-3’ |
|  | Reverse: 5’-CAGGTCCCAGCGCAATGTAAC-3’ |
| OPG | Forward: 5’-AGCAGGAGTGCAACCGCACC-3’ |
|  | Reverse: 5’-TTCCAGCTTGCACCACGCCG-3’ |
| TLR2 | Forward: 5’-CCCTTCTCCTGTTGATCTTGCT-3’ |
|  | Reverse: 5’-CGCCCACATCATTCTCAGGTA-3’ |
| TLR4 | Forward: 5’-GCAGAAAATGCCAGGATGATG-3’ |
|  | Reverse: 5’-TCTGATCCATGCATTGGTAGGT-3’ |
| IRAK1 | Forward: 5’-GAGACCCTTGCTGGTCAGAG-3’ |
|  | Reverse: 5’-GCTACACCCACCCACAGAGT-3’ |
| TRAF6 | Forward: 5’-GCCCAGGCTGTTCATAATGT-3’ |
|  | Reverse: 5’-CGGATCTGATGGTCCTGTCT-3’ |
| NAMPT | Forward: 5’-CCTTTTGTCATTAATCAGC-3’ |
|  | Reverse: 5’-TTTGGCATCACTGGTACATA-3’ |
| SIRT1 | Forward: 5’-ATGACGTCTTGTCCTCTAGT-3’ |
|  | Reverse: 5’-CTCTCCGTATCATCTTCCAA-3’ |
| β-actin | Forward: 5’-GCAGGAGTACGATGAGTCCGG-3’ |
|  | Reverse: 5’-CTTTGGGGGATGTTTGCTCCA-3’ |

**
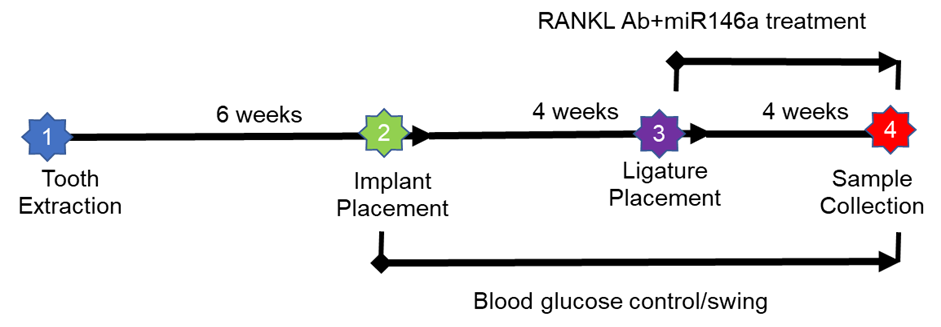
**

**Supplemental FigureS1.Procedures of implantation, ligation, blood glucose control and treatment.**1, Maxillary first and second molars were extracted; 2. Six weeks post-extraction, the implant was placed; *Rosiglitazoneadministration was used to control blood glucose level.*3. Four weeks after implant placement, the silk ligature was placed around implant; Gingival RANKL Ab and miR146a injection was performed. 4. Four weeks after ligation, mice were euthanized and samples were collected.

**A**


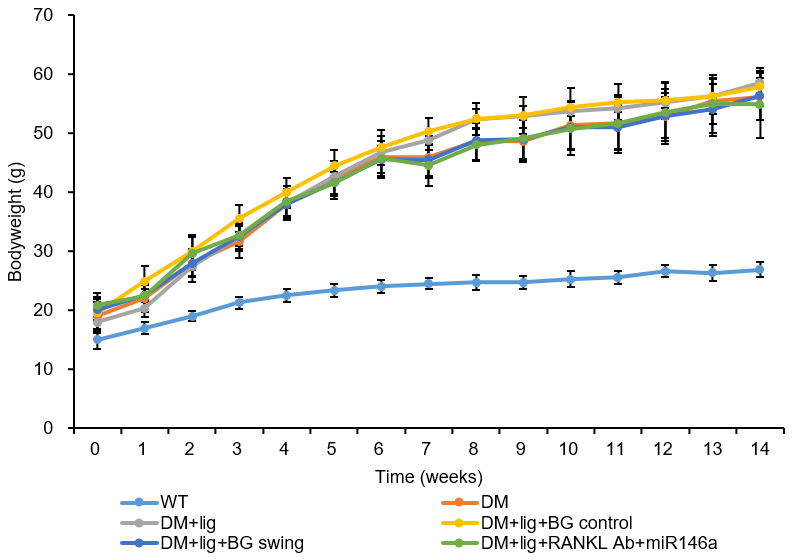


**B**


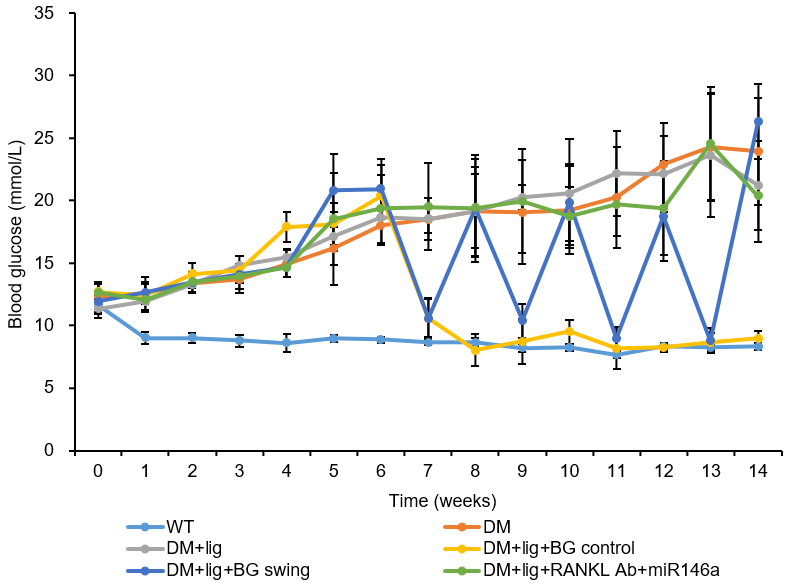


**Supplemental FigureS2. Bodyweight and fasting blood glucose of different groups of mice**. (**A**) Bodyweight of mice was measured every week until sacrifice (Mean ± SD, n=6). (**B**) Fasting blood glucose of mice was detected every week until sacrifice (Mean ± SD, n=6).


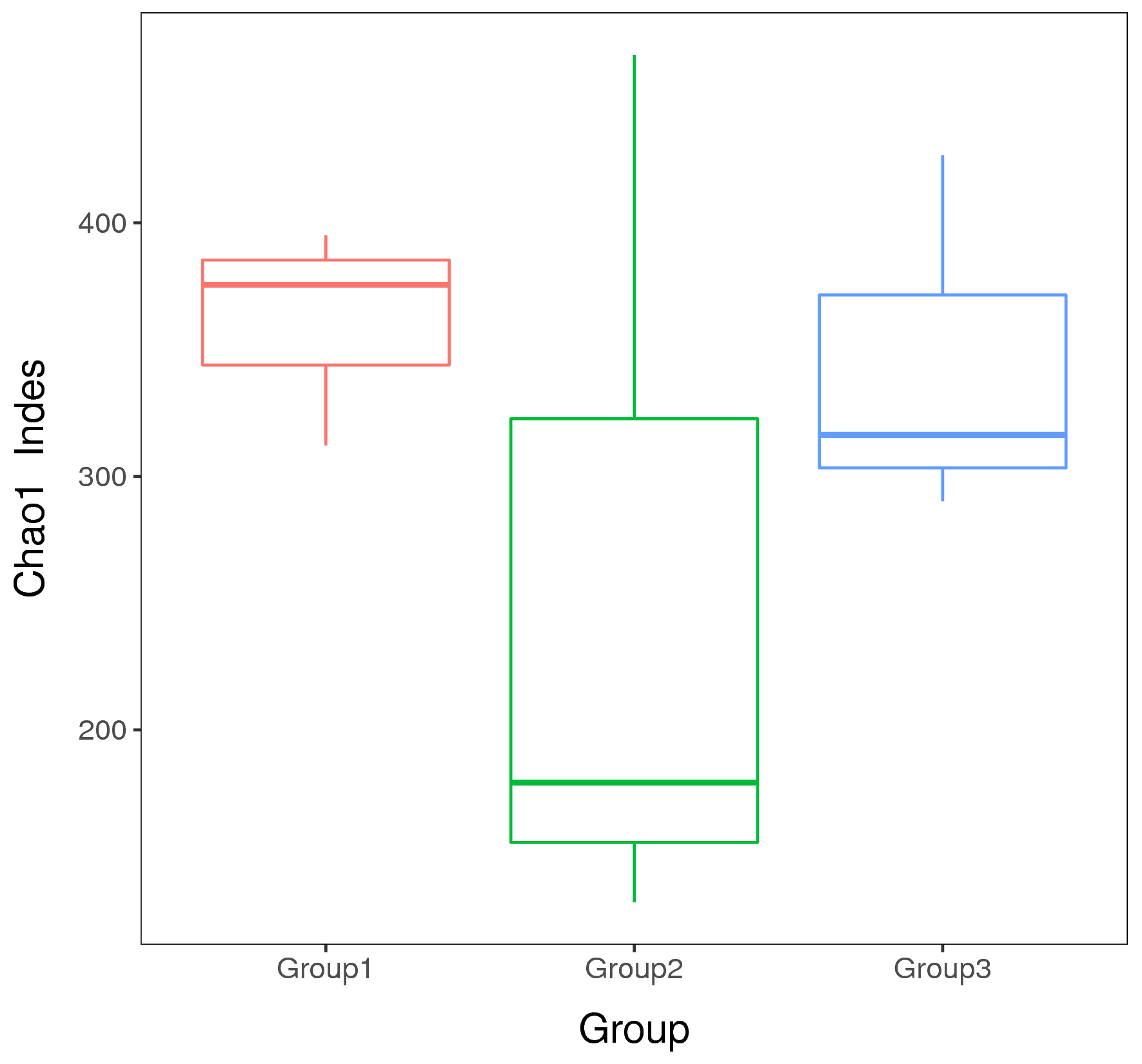


**DM+lig**

**DM+lig**

**+BG control**

**DM+lig**

**+BG swing**


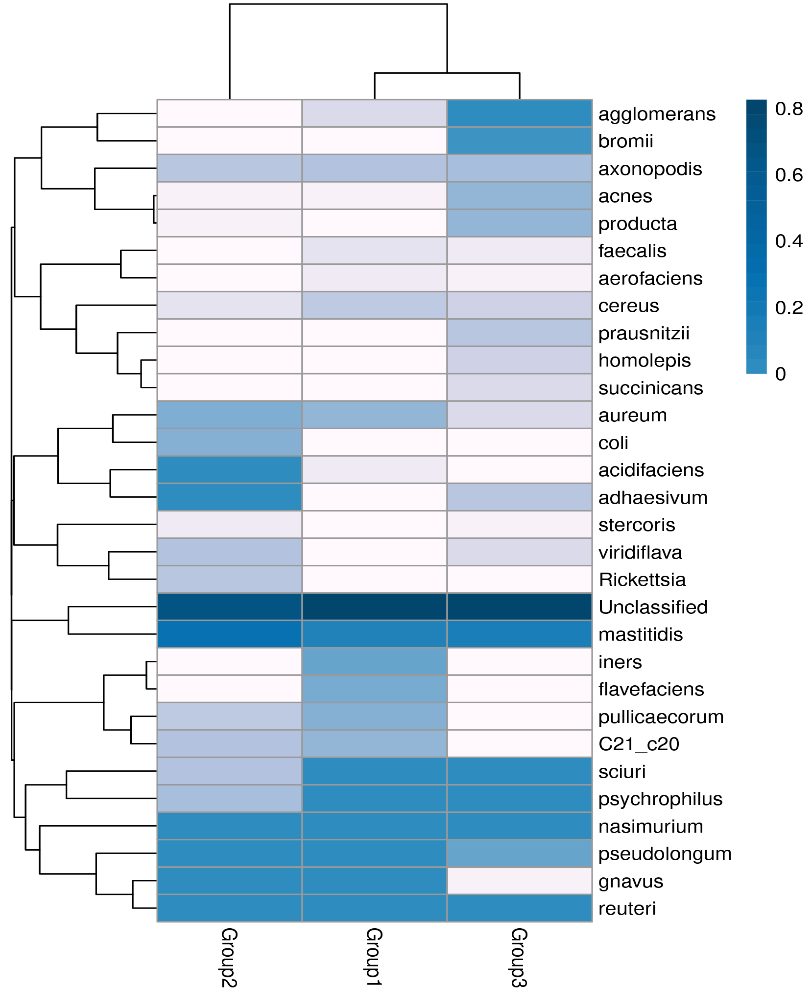


**DM+lig**

**DM+lig**

**+BG control**

**DM+lig**

**+BG swing**

**A**

**B**

**Supplemental FigureS3.Species of ligation silk bacteria were analyzed using 16S rRNA gene sequence**. The bacterial composition from ligation silks of different groups of mice was analyzed using 16S rRNA gene sequence. (A) Chao1 index of bacterialcommunity from ligation silks of DM+lig, DM+lig+BG control and DM+lig+BG swing groups (Mean ± SD, n = 3); (B) The heatmap of species abundance in DM+lig, DM+lig+BG control and DM+lig+BG swing groups.
